# Lithium Intoxication in an Elderly Woman

**DOI:** 10.7759/cureus.32997

**Published:** 2022-12-27

**Authors:** Sara Gomes, Ines Bonito, Sara Santos, Monica Silva, Teresinha Ponte

**Affiliations:** 1 Internal Medicine Department, Centro Hospitalar Barreiro Montijo, Barreiro, PRT

**Keywords:** acute kidney injury, acute confusional state, lithium toxicity, lithium intoxication, delirium

## Abstract

Acute confusional state or delirium in the elderly frequently requires a lengthy differential diagnosis in the emergency room (ER) to determine the factors of its multiple causes. Iatrogeny can be one of the causes, especially in elderly people with polypharmacy. We present a case of a 77-year-old female, independent in activities of daily living, with no cognitive impairment and a history of hypertension, dyslipidemia, and manic-depressive disorder. She arrived at the ER with diarrhea, vomiting, and myalgias. A blood test revealed an acute kidney injury. The patient was discharged with the diagnosis of acute gastroenteritis and prerenal acute kidney injury.

The patient returned to the ER two days later due to worsening symptoms, including spatial and temporary disorientation and a marked prostration. The attending physician recommended a lithium blood level test due to the patient's history and the outpatient's psychiatric medication. The tests revealed a value of 2.18 mmol/L (toxic levels: >2.0 mmol/L). Support measures were initiated with diuresis control and vigorous hydration, with subsequent clinical and biochemical improvement (lithium blood levels reduced to 0.97 mmol/L).

Lithium toxicity causes acute nausea, vomiting, diarrhea, and neurological symptoms that have a slower onset and correlate with chronic toxicity. A declining renal function and reduced volume of distribution (due to increased body fat mass and decreased total body water) contribute to more significant pharmacological toxicity in the elderly. In this case, dehydration triggered by diarrhea and vomiting may have been a cause or a consequence. Reviewing chronic medication and a detailed investigation of all etiological causes was essential for the patient's rehabilitation, avoiding possible irreversible neurological damage.

## Introduction

An acute confusional state or delirium is one of the most frequent conditions in the emergency room (ER). This condition accounts for about 30% of hospitalized patients, mainly among the elderly [1]. Delirium is a multifactorial condition in which risk factors can be divided into those vulnerable to delirium and those that precipitate it. About half of the patients with delirium who are most susceptible include those with underlying central nervous system diseases such as dementia, stroke, or Parkinson's disease [2]. Also, advanced age and sensory impairment increase this risk. The precipitating factors of delirium are varied, and multiple pharmacological classes may be responsible: antibiotics and antivirals, anticholinergics, anticonvulsants, antidepressants, some antiarrhythmic agents, antihypertensives and corticosteroids, and among others. From this immense variety of possible factors, we have lithium. This drug, used since the middle of the 20th century [3] for disorders such as bipolar affective disorder, recurrent depressive disorder, cluster headaches, migraines, and impulsive behavior, is recurrently used in clinical practice, making it essential to recognize its possible adverse effects.

Given the frequency and diversity of etiologies of delirium in the geriatric population, diagnosis is sometimes difficult. Often, a complete etiological study is not carried out, assuming that the most frequent causes in this population, such as dehydration, infection, or the simple presence in a hospital environment, are solely responsible. There is also a tendency to assume that the senior adult shows signs of an early stage of dementia, which, despite being more frequent in this population, also deserves a pertinent study and differential diagnosis. An example of this is the clinical case described below.

## Case presentation

A 77-year-old female with a personal history of arterial hypertension, dyslipidemia, and bipolar disorder was admitted to the ER due to diarrhea and myalgias with four days of evolution and vomiting with two days of development, with no other symptoms (namely neurological). She did not know how to specify her usual medication. The objective examination had no relevant alterations, and she was conscious, oriented, and hydrated. Urinary output was 1.5 mL/kg/h. Analytically, she presented with an acute kidney injury (creatinine 1.69 mg/dL-AKIN 1) with no other changes (Table 1, first column). Given the clinical stability, she was discharged with the diagnosis of acute gastroenteritis with subsequent prerenal acute kidney injury. The assisting physician recommended the patient increase her water intake.

**Table 1:** Creatinine, urea, and lithium levels in the first admission to the ER (September 27, 2018). On the patient's return (September 29, 2018) and after the start of treatment (October 1, 2018), with respective reference range levels. ER: emergency room.

	First admission to ER	Second admission to ER	Third day of hospitalization	Reference range levels
Creatinine	1.69 mg/dL	2.01 mg/dL	1.0 mg/dL	0.55–1.02 mg/dL
Urea	55 mg/dL	63 mg/dL	35 mg/dL	10–50 mg/dL
Lithium	-	2.2 mmol/L	0.97 mmol/L	0.6–1.2 mmol/L

After two days, the patient returned to the ER due to the maintenance of symptoms, temporal-spatial disorientation, and marked prostration. Upon observation, she presented with sunken-appearing eyes and decreased skin turgor but was normotensive, normocardic and with capillary refill time under three seconds. The patient responded after vigorous stimulation but was unable to sustain attention. With regards to language, she said a few sentences but needed repeated stimulation to carry out simple orders. The pupils were symmetrical and reactive. Oculomotor movements were intact. The face was symmetrical, with the tongue in the midline. Motor and sensory assessment were limited given the inability to follow orders, but the patient moved all four extremities equally and spontaneously and reacted to pain in all extremities. The first diagnostic hypothesis considered was acute confusional syndrome in the context of dehydration. An early stage of dementia (given the age) was also considered.

Given the personal history of bipolar disorder, her prescription history was revised, confirming prescriptions for lithium. Serum lithium levels were requested, showing a value of 2.2 mmol/L (Table 1, second column). Concerning the remaining exams, the renal function was aggravated (Table 1, second column). There were no other analytical changes, including white blood cells, c-reactive protein, urinalysis, thyroid function, and B12 vitamin. A computed tomography (CT) scan of the head, thorax radiography, and an electrocardiogram were also normal. Given that the performed exams were unremarkable, lithium intoxication was the most likely hypothesis.

Poison information centers and dialysis centers were contacted, with an indication for exploitation through vigorous hydration. After three days of vigorous hydration (3 l/day) and diuresis control (2 ml/kg/h), serum lithium level decreased by 1.23 mmol/L, glomerular filtration rate (Chronic Kidney Disease Epidemiology Collaboration (CKD-EPI) equation) increased from 25 ml/min/1.73 m^2^ to 58 ml/min/1.73 m^2^, and the patient was conscious and oriented. Table 1 shows the evolution of renal function and serum lithium levels. The patient was discharged after five days of hospitalization.

## Discussion

According to Haussmann et al., the effective dose of lithium is 0.6-1 mmol/L in prolonged administration, values above 1.2 mmol/L may be toxic [4]. Despite being a widely used drug, the mechanism of lithium neurotoxicity is still poorly understood and can sometimes occur at therapeutic levels [3,5]. It's a drug with a narrow therapeutic index [6-8], and the relationship between dose and adverse effects is not yet defined [6,9]. The risk of severe neurotoxicity is thought to be greater in patients on chronic medication [3]. Other known risk factors are age over 50, kidney damage, and thyroid disease [3,9].

Lithium toxicity is divided into acute, acute-on-chronic, and chronic [6,7]. Acute intoxication is usually associated with an episode of heavy ingestion, and given the absorption of lithium from the gastrointestinal tract, the primary symptoms are gastrointestinal [7]. Chronic intoxications are more frequent due to mechanisms that reduce renal lithium expoliation, such as dehydration, infection, or drug interactions [6,7]. Patients with acute-on-chronic ingestion usually present signs and symptoms of both previously mentioned intoxications [7].

As mentioned, initial symptoms are usually gastrointestinal: diarrhea, nausea, and vomiting. With decreased concentration, moderate ataxia and weakness may also be present. Furthermore, with increased toxicity, neurological symptoms prevail, evolving into coarse tremors, slurred speech, confusion, mood changes, memory, and front-to-executive dysfunction, which can culminate in seizures and stupor [3,4,6]. Neurotoxicity can be reversible or irreversible, and cerebellar signs, such as ataxia and dysarthria, are usually associated with irreversibility [3]. The longer the intoxication, the greater the likelihood of irreversible brain damage.

A lower glomerular filtration rate and different volume distribution contribute to greater pharmacological toxicity in the elderly. In addition, lithium-associated nephropathy, in which focal nephron atrophy and interstitial fibrosis are observed [6], is another aggravating factor. In this case, dehydration triggered by diarrhea and vomiting may have been a cause or a consequence.

Regarding the diagnostic tests, in addition to serum lithium levels above average values, diffuse slowing can also be observed in the electroencephalogram (during acute neurotoxicity), and the magnetic resonance imaging and computerized tomography scans are often normal [3].

Treatment guidelines vary according to the degree of intoxication, ranging from drug discontinuation to extracorporeal methods [6]. Under the recommendations of the US extracorporeal treatments in poisoning, since lithium is a dialyzable substance, extracorporeal therapy is recommended if lithium values exceed 4.0 mmol/L or in the presence of decreased consciousness, convulsions, or life-threatening dysrhythmias. These therapies should be continued until values lower than 1.0 mmol/L are reached [3]. The US guidelines suggest initiating this dialytic therapy after marked ingestion of lithium even without symptoms [3], although the timing of initiation of renal replacement therapy is not yet defined. In patients with concentrations of 2.5 mmol/L and signs of severe intoxication, hemodialysis should also be started [6]. It should always be considered that the elimination half-life of lithium is between 12 and 48 hours in patients with chronic therapy, with the elderly having longer half-lives [7]. Due to the lack of correlation between serum lithium levels and clinical severity, and the insufficient and inconsistent data regarding hemodialysis indications, the therapeutic approach should be carried out on a case-by-case basis, focusing on the patient's status [6,7].

## Conclusions

In retrospect, the discharge of a patient with kidney damage on lithium therapy excreted by the kidney was troublesome. Confirmation of chronic medication and investment in the etiological study was essential for the patient's rehabilitation, avoiding possible irreversible neurological damage. It is vital to review and control therapies in elderly patients since they are more prone to iatrogenesis. Educating the family and patients about the adverse effects of treatments and prodromal symptoms of intoxication, informing patients about the importance of knowing their outpatient therapy (namely psychiatric), and performing a complete history collection are essential tools that minimize risks and fatalities.
